# Longitudinal Study of Treatment Variability for Parkinson's Disease across Specialized Centers

**DOI:** 10.1002/mdc3.70232

**Published:** 2025-07-15

**Authors:** Nabila Dahodwala, Theodore Kapogiannis, Amanda Cruz, James C. Beck, Thomas L. Davis, Hongliang Liu, Sheng Luo, Anna Naito, Marilyn Neault, Miriam R. Rafferty, Adolfo Ramirez‐Zamora, Connie Marras

**Affiliations:** ^1^ Department of Neurology, Parkinson's Disease and Movement Disorder Center University of Pennsylvania Philadelphia Pennsylvania USA; ^2^ Parkinson's Foundation New York New York USA; ^3^ Department of Neurology Vanderbilt University Nashville Tennessee USA; ^4^ Department of Population Health Sciences Duke University School of Medicine Durham North Carolina USA; ^5^ Department of Biostatistics & Bioinformatics Duke University Durham North Carolina USA; ^6^ Person with Parkinson's, Parkinson's Foundation New York New York USA; ^7^ Department of Physical Medicine and Rehabilitation, Shirley Ryan AbilityLab; Northwestern University Department of Physical Medicine and Rehabilitation Chicago Illinois USA; ^8^ Department of Neurology The Norman Fixel Institute for Neurological Diseases at the University of Florida Gainesville Florida USA; ^9^ Department of Neurology, The Edmond J Safra Program in Parkinson's Disease University Health Network, University of Toronto Toronto Canada

**Keywords:** real‐world evidence, best practices, quality of care, centers of excellence

## Abstract

**Background:**

Real‐world evidence on treatment practices in Parkinson's disease (PD) has been limited due to the difficulty in collecting comprehensive and generalizable clinical data.

**Objectives:**

We sought to identify treatment patterns and test how treatment changed in response to (1) falling, (2) worsening disease, and (3) worsening quality of life across PD specialized centers.

**Methods:**

We used the Parkinson Outcomes Project data collected from 2010 to 2023 across 31 international PD specialized centers. Demographic and clinical characteristics were collected annually and included medication use, physical therapy referral, psychologist or psychiatrist care, and deep brain stimulation (DBS) surgery. Treatment practice variation was described by center and in response to outcomes (self‐reported falls, higher Hoehn and Yahr stage, worse emotional and mobility subscale scores on quality‐of‐life scale).

**Results:**

A total of 12,664 participants were analyzed. Treatment practices varied substantially across centers with the use of levodopa in the first 5 years of disease ranging from 59.3% to 94.6% and physical therapy referral ranging from 13% to 71%. At ≥ 5 years of disease, DBS rates varied from 2% to 41%. After a fall, individuals were more likely to be referred for physical therapy (*β*: 0.44, 95% confidence interval [CI]: 0.36, 0.52), and mental health services were recommended after a decline in emotional subscores (*β*: 1.74, 95% CI: 1.50, 1.98). However, there was no change in levodopa‐equivalent daily dose after worsening mobility subscores (*β*: −29.97, 95% CI: −76.67, 16.73).

**Conclusions:**

These results highlight the large variability in PD practice across specialty centers and the importance of establishing best practice guidelines. Understanding the drivers of this variability is an essential next step.

The treatment of Parkinson's disease (PD) is primarily informed by anecdotal experience and randomized controlled clinical trials (RCT), which can have strict inclusion/exclusion criteria and relatively small sample sizes. RCTs tend to recruit subjects who are younger, with higher educational attainment and have fewer comorbidities than the average PD patient, and samples in these studies do not adequately capture the clinical heterogeneity of the PD population.^1^, ^2^, ^3^ Consequently, findings from RCTs lack generalizability, and it remains unclear how this evidence translates to the real‐world management of PD.^4^

To bridge this gap, the collection of real‐world data on the treatment of PD is necessary. Studies using real‐world data can serve as extensions of RCTs, evaluating how interventions influence PD outcomes in larger and more representative cohorts. Furthermore, real‐world data can be used to describe the current treatment practices in PD and offer insight into how they differ from guideline‐based strategies. Real‐world data are challenging to procure, though, and most clinical practice databases in PD lack sufficient statistical power for research and are derived from a single center. As a result, prior research describing treatment practices has largely relied on the analysis of medical claims.^5^, ^6^ However, these studies are limited by lack of granular clinical data and potential miscoding and misclassification of individuals.

The Parkinson's Outcomes Project (POP) is a prospective, observational study collecting real‐world data on treatments and outcomes from more than 12,000 individuals with PD across 31 international Parkinson's Foundation Centers of Excellence (COE) with the goal of providing insights to improve the quality and delivery of PD care.^1^, ^7^ Briefly, COEs are academic medical centers that meet established criteria, which include seeing 700 unique PD patients annually and providing exemplary team care, comprising a neurologist with training in movement disorders, nurse, social worker, physical therapist, occupational therapist, and speech‐language pathologist. In this study, we analyzed a unique dataset and hypothesized that significant variation would be observed in treatment practices across COEs. We tested if physical therapy (PT) referral is predicted by falls or worsening Hoehn and Yahr (H&Y) stage, if mental health services referral is predicted by worse emotional subscale scores on the Parkinson's Disease Questionnaire‐39 (PDQ‐39), and if change in levodopa‐equivalent daily dose (LEDD) is predicted by worse PDQ‐39 mobility subscale or H&Y stage.

## Methods

### Study Design and Sample

This was a retrospective cohort study using data collected prospectively from the Parkinson Foundation's Parkinson's Outcomes Project (PF‐POP). The PF‐POP is an observational, longitudinal study of treatment practices and health outcomes in PD that collected data annually from patients during their regularly scheduled clinic visits at PF Centers of Excellence.^8^ Data were collected from 2010 to 2023. Participants were recruited as a convenience sample through clinic referrals. The only inclusion criterion was a physician diagnosis of PD. For analyses of baseline data, at least 1 study visit was required, and in analyses of practice variation following treatment, at least 2 study visits were required. A total of 31 global centers (22 North American, 8 European, and 1 Middle Eastern) participated in this study (Fig. S1).

The study received approval from each of the participating centers' individual Institutional Review Boards (IRBs), and written informed consent was obtained from all participants. Data are reported in aggregate, and centers are deidentified.

### Measures

Data collected from patients included demographics and clinical data on disease characteristics, treatments, and outcomes. Disease characteristics included disease duration, H&Y stage,^9^ and medical comorbidities. Specifically, we asked participants if they had high blood pressure, heart disease, anemia or other blood disease, lung disease, cancer, diabetes, ulcer/stomach disease, constipation, liver disease, kidney disease, depression, psychosis, osteoarthritis/degenerative arthritis, back pain, and rheumatoid arthritis. Participants could report up to four additional diseases not captured by the list (e.g., skin cancer, stroke). Treatment practices included medication use with total dose (in mg), use of other therapies (such as PT, psychologist, or psychiatric care), and deep brain stimulation (DBS) surgery. LEDD was calculated based on published conversion factors for levodopa formulations, catechol‐*O*‐methyl transferase (COMT) inhibitors, monoamine oxidase‐B (MAO‐B) inhibitors, dopamine agonists, and amantadine.^10^ Outcomes were measured using the PDQ‐39, where higher scores indicate worse quality of life.^11^ Previously, researchers have shown the minimal clinically important difference for the PDQ‐39 summary index (0–100) to be ±4.72, and there is a suggestion that a similar difference may apply to transformed subscale scores.^12^, ^13^ Both the mobility subscore (0–40) and emotional well‐being subscore (range 0–24) were analyzed separately. Falls in the 3 months prior to the visit were also assessed.

### Analysis

Variation in treatment practices at the baseline visit was first described by center and stratified by disease duration (< 5 years vs. ≥ 5 years). We chose this cutoff to approximate early versus moderate to advanced PD stages based on prior literature.^14^ We applied a logistic regression to compare the proportion of individuals taking one treatment (ie, using levodopa, dopamine agonist, PT, psychiatrist/psychologist, or DBS) in one individual center with the average across all centers after adjusting for patient age, sex, education, and number of comorbidities. Next, treatment practices at visit 2 were analyzed in response to specific key issues present at visit 1 for individuals: (1) PT utilization after a fall; (2) mental health use if PDQ‐39 emotional subscale score was > 9 based on prior analyses showing strong correlation with other validated markers of depression^15^; (3) change in LEDD and PT utilization in those with a change in H&Y stage from 1/2 to 3 to 4/5 H&Y stage; (4) change in LEDD for PDQ‐39 mobility subscale score > 11, which was the median value across patients at all centers. We applied a generalized linear mixed model to test the association between clinical characteristics in previous visit and clinical practices in next visit. Models included baseline age, sex, PD duration, and H&Y stage (if not the predictor variable) as covariates, and the random effects of centers and individuals. Treatment practice variation is also reported by center and by individual. Missing data for covariates were managed with multiple imputation using the R package mice. For missing data on dependent variables, we used complete case analysis by excluding observations with missing values. Our manuscript is structured in accordance with the Strengthening the Reporting of Observational Studies in Epidemiology (STROBE) criteria.^16^

## Results

The cohort selected for this analysis included 12,664 PD patients across 31 COEs. Table 1 describes demographic and clinical characteristics of this sample stratified by center. For the demographic and clinical variables included in Table 1, 9.1% of data were missing. At baseline, the mean (standard deviation [SD]) disease duration was 7.1 years (±5.8), with 6463 (51%) participants having a disease duration >5 years. The mean (SD) age was 68 (±10), with 63% identifying as male and 93% as White.

**TABLE 1:** Demographic and clinical characteristics of patients (N = 12,664) by center at baseline visit (n = 31)

Center #	N	Age, mean (SD)	Male, n (%)	White, n (%)	Non‐Hispanic, n (%)	Level of education, n (%)				Disease duration		
						≤12 yr	13–14 yr	15–16 yr	≥16 yr	Mean (SD)	≤5 yr, n (%)	>5 yr, n (%)
1	573	67.1 (9.8)	386 (67.4)	498 (96.5)	485 (94.4)	34 (18)	44 (23.3)	50 (26.5)	61 (32.3)	8.5 (6)	211 (36.8)	362 (63.2)
2	495	69.6 (10.1)	305 (61.6)	433 (89.1)	481 (99.2)	5 (29.4)	3 (17.6)	5 (29.4)	4 (23.5)	7.8 (5.2)	205 (41.4)	290 (58.6)
3	667	69.2 (9.5)	400 (60)	634 (97.4)	638 (99.5)	30 (8.7)	108 (31.3)	116 (33.6)	91 (26.4)	6.8 (5.2)	319 (47.8)	348 (52.2)
4	585	66.3 (9.9)	369 (63.1)	553 (96.5)	561 (98.6)	15 (6.6)	69 (30.5)	64 (28.3)	78 (34.5)	7.8 (6.4)	276 (47.2)	309 (52.8)
5	599	68 (10.2)	366 (61.2)	529 (91.2)	550 (94.8)	30 (15.5)	27 (13.9)	58 (29.9)	79 (40.7)	5.7 (5.2)	352 (58.8)	247 (41.2)
6	599	66.2 (9.5)	363 (60.6)	561 (94.4)	584 (99.3)	28 (13.1)	40 (18.8)	65 (30.5)	80 (37.6)	7.9 (6)	252 (42.1)	347 (57.9)
7	705	65.9 (9.5)	455 (64.6)	624 (89.9)	619 (89.1)	30 (11.2)	62 (23.2)	93 (34.8)	82 (30.7)	6.9 (6)	361 (51.2)	344 (48.8)
8	444	66.1 (9.8)	294 (66.2)	425 (96.4)	421 (99.1)	20 (10.8)	29 (15.6)	53 (28.5)	84 (45.2)	6.9 (6.2)	237 (53.4)	207 (46.6)
9	726	66.6 (9.4)	475 (65.4)	715 (98.8)	710 (97.9)	108 (21.3)	114 (22.4)	155 (30.5)	131 (25.8)	7.2 (5.4)	341 (47)	385 (53)
10	632	66.4 (9.5)	404 (63.9)	588 (93.2)	611 (97.4)	47 (14.8)	54 (17)	81 (25.5)	136 (42.8)	5.6 (5.6)	376 (59.5)	256 (40.5)
11	418	70.1 (9.8)	268 (64.1)	372 (91)	363 (92.6)	54 (31.4)	32 (18.6)	33 (19.2)	53 (30.8)	7.1 (5.6)	206 (49.3)	212 (50.7)
12	494	67.9 (9.3)	301 (60.9)	463 (93.9)	459 (98.9)	45 (26.3)	49 (28.7)	39 (22.8)	38 (22.2)	6.8 (6)	255 (51.6)	239 (48.4)
13	264	69.4 (9.2)	181 (68.6)	209 (89.3)	146 (89.6)	4 (3.3)	12 (9.8)	55 (44.7)	52 (42.3)	7.7 (5.7)	119 (45.1)	145 (54.9)
14	584	69 (9.8)	363 (62.2)	544 (94.3)	436 (93.8)	13 (20.3)	14 (21.9)	25 (39.1)	12 (18.8)	5.8 (5.5)	345 (59.1)	239 (40.9)
15	556	65.2 (8.3)	353 (63.5)	541 (97.3)	551 (99.3)	83 (25.5)	70 (21.5)	100 (30.8)	72 (22.2)	7.3 (5.9)	261 (46.9)	295 (53.1)
16	106	67.1 (9.9)	62 (58.5)	87 (87)	100 (95.2)	‐	‐	‐	‐	8.2 (6.8)	47 (44.3)	59 (55.7)
17	375	68 (9.2)	239 (63.7)	355 (94.9)	353 (95.7)	23 (12.6)	47 (25.8)	59 (32.4)	53 (29.1)	6.3 (5.3)	202 (53.9)	173 (46.1)
18	643	68.3 (10.1)	428 (66.6)	643 (100)	641 (100)	97 (36.1)	79 (29.4)	26 (9.7)	67 (24.9)	8.2 (6.5)	273 (42.5)	370 (57.5)
19	199	65.1 (10.4)	130 (65.7)	193 (98.5)	153 (98.7)	8 (80)	1 (10)	1 (10)	0 (0)	4.3 (4.7)	135 (67.8)	64 (32.2)
20	562	65.6 (9.4)	378 (67.4)	496 (88.6)	550 (98.9)	51 (13.9)	104 (28.3)	97 (26.4)	116 (31.5)	7.6 (5.9)	249 (44.3)	313 (55.7)
21	318	66.6 (9.7)	200 (62.9)	277 (87.9)	114 (40)	55 (23.1)	55 (23.1)	72 (30.3)	56 (23.5)	6.3 (5.4)	179 (56.3)	139 (43.7)
22	241	67.4 (9.9)	145 (60.2)	230 (95.4)	237 (98.8)	65 (27)	70 (29)	61 (25.3)	45 (18.7)	6.1 (5.4)	126 (52.3)	115 (47.7)
23	197	68.1 (9.4)	114 (57.9)	183 (94.3)	188 (96.9)	12 (6.1)	29 (14.7)	57 (28.9)	99 (50.3)	6.2 (5.1)	114 (57.9)	83 (42.1)
24	210	68.7 (9.3)	132 (65)	197 (93.8)	209 (99.5)	75 (35.7)	67 (31.9)	35 (16.7)	33 (15.7)	9.3 (6.6)	76 (36.2)	134 (63.8)
25	210	69.9 (9.7)	117 (55.7)	208 (99)	209 (99.5)	158 (75.6)	20 (9.6)	19 (9.1)	12 (5.7)	6.1 (5.3)	121 (57.6)	89 (42.4)
26	357	68.9 (8.8)	217 (61.3)	355 (99.7)	18 (5.1)	276 (78.4)	17 (4.8)	38 (10.8)	21 (6)	9.3 (5.6)	101 (28.3)	256 (71.7)
27	202	67.8 (11.7)	113 (55.9)	189 (95)	199 (98.5)	144 (71.6)	21 (10.4)	12 (6)	24 (11.9)	5.6 (5.2)	124 (61.4)	78 (38.6)
28	205	71.4 (9.5)	129 (62.9)	204 (99.5)	204 (99.5)	186 (90.7)	5 (2.4)	1 (0.5)	13 (6.3)	6 (6)	118 (57.6)	87 (42.4)
29	197	69.4 (8.7)	122 (61.9)	197 (100)	197 (100)	170 (86.7)	1 (0.5)	9 (4.6)	16 (8.2)	10.1 (5.7)	45 (22.8)	152 (77.2)
30	200	68.8 (9.6)	123 (61.5)	200 (100)	200 (100)	167 (83.5)	3 (1.5)	4 (2)	26 (13)	6.1 (5.3)	115 (57.5)	85 (42.5)
31	101	68.7 (8.7)	69 (68.3)	77 (87.5)	82 (83.7)	12 (12)	17 (17)	30 (30)	41 (41)	6.2 (5.4)	60 (59.4)	41 (40.6)
Total	12,664	67.6 (9.7)	8001 (63.2)	11,780 (94.6)	11,269 (93.0)	2045 (31.5)	1263 (19.4)	1513 (23.3)	1675 (25.8)	7.1 (5.8)	6201 (49.0)	6463 (51.0)

*Note*: (−) indicates missing data.

Abbreviation: SD, standard deviation.

**Fig. 1:**
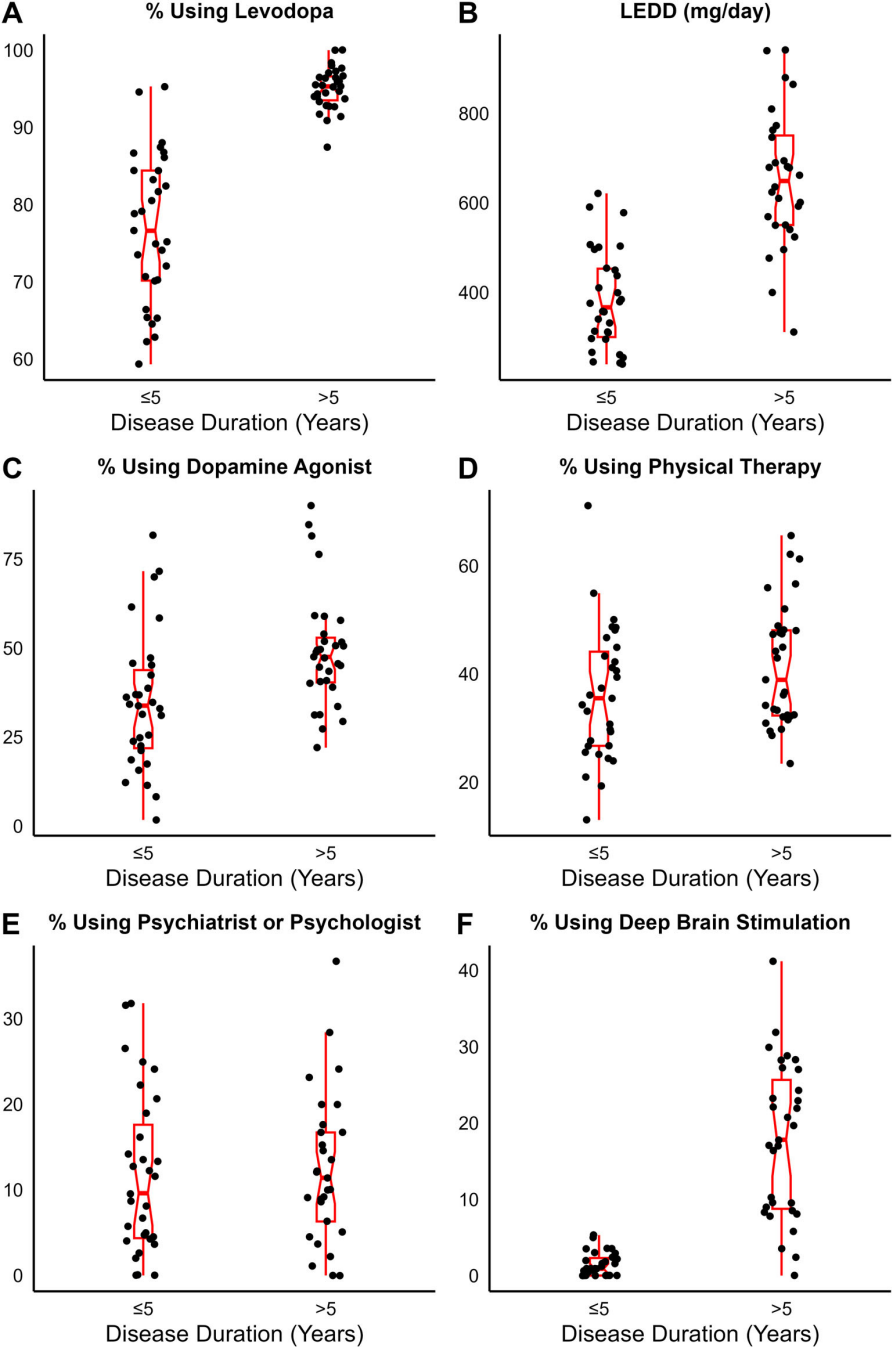
Utilization of (**A**) levodopa, (**B**) levodopa‐equivalent daily dose, (**C**) dopamine agonist, (**D**) physical therapy, (**E**) psychiatrist or psychologist, (**F**) deep brain stimulation for patients with disease duration of ≤5 and >5 years by center adjusted for and comorbidities, age, sex, level of education.

The utilization of primary treatment practices, by center, is shown in Figure 1. Across treatment practice variables included in Figure 1, 23.8% of data was missing. For each treatment practice, the median frequency or dose was higher for participants with disease duration >5 years relative to those with disease duration ≤5 years. Mean LEDD varied across centers, ranging from 239 to 621 mg for participants with disease duration ≤5 years and 311–941 mg for disease duration >5 years. Dopamine agonist use varied widely, ranging from 2% to 82% of participants with disease duration ≤5 years and 22% to 90% with disease duration >5 years. Center referral rates to PT for participants with disease duration ≤5 ranged from 13% to 71%, with 9 centers referring significantly more (*P* < 0.05) and 8 centers less (*P* < 0.05) than average. Center referral rates for PT for participants with disease duration >5 ranged from 23% to 66%, with 9 centers referring more (*P* < 0.05) and 10 centers less (*P* < 0.05) than average. For participants with disease duration ≤5 years, levodopa use ranged from 82% to 95% across centers, with 7 centers using levodopa more (*P* < 0.05) and 10 centers less (*P* < 0.05) than average. For participants with disease duration >5 years, DBS use ranged from 2% to 41% across centers, with 12 centers using DBS more (*P* < 0.05) and 8 centers less (*P* < 0.05) than average. The data on treatment practice utilization by center are presented in Tables S1 and S2.

**Fig. 2:**
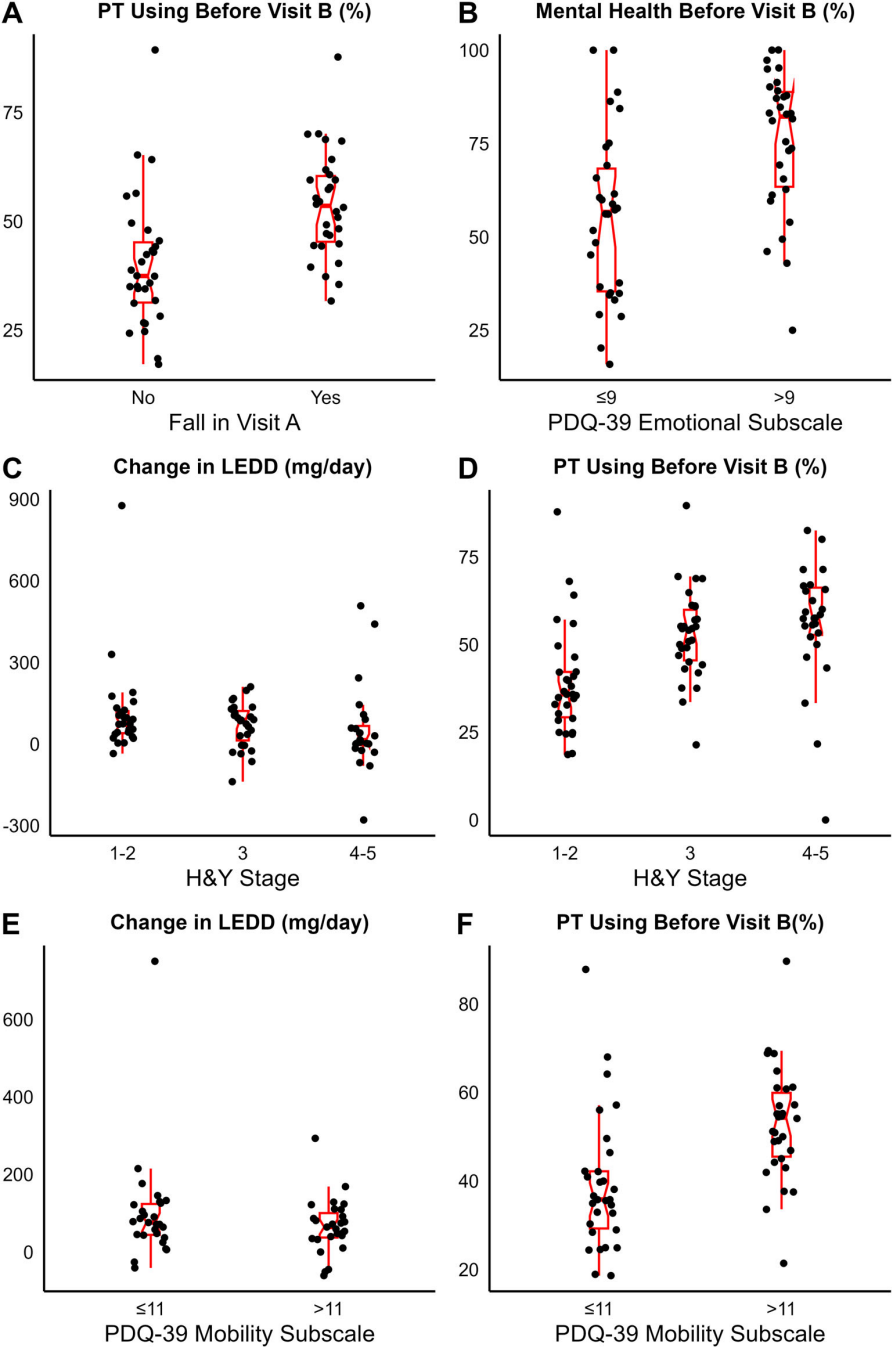
Utilization of treatment practices in the follow‐up visit (visit B) stratified by key clinical characteristics in the initial visit (visit A).

The utilization of treatment practices in the follow‐up visit, stratified by key clinical characteristics identified in the initial visit, is shown in Figure 2. Center 31 was excluded from Table 2 as follow‐up data were unavailable. Mean utilization of mental health services and PT were higher for participants with worse clinical characteristics.

**TABLE 2:** Association between key clinical characteristics in initial visit and treatment practices in follow‐up visit

Outcome	Predictor	*β*	95% CI	*P*
PT using in next visit	Falls (yes vs. no)	0.44	(0.36, 0.52)	<0.001
Mental health services	PDQ‐39 emotional subscale >9 vs. ≤9	1.74	(1.50, 1.98)	<0.001
Change in LEDD	H&Y Stage 3 vs. 1–2	−34.50	(−91.74, 22.73)	0.24
	H&Y stage 4–5 vs. 1–2	−63.23	(−155.34, 28.87)	0.18
PT using in next visit	H&Y Stage 3 vs. 1–2	0.47	(0.38, 0.56)	<0.001
	H&Y stage 4–5 vs. 1–2	0.62	(0.47, 0.76)	<0.001
Change in LEDD	PDQ‐39 mobility subscale >11 vs. ≤11	−29.97	(−76.67, 16.73)	0.21
PT using in next visit	PDQ‐39 mobility subscale >11 vs. ≤11	0.47	(0.39, 0.55)	<0.001

*Note*: Generalized linear mixed model included age, sex, PD duration, H&Y stage, and the random effects of centers and individuals. (−) indicates missing data.

Abbreviations: CI, confidence interval; PT, physical therapy; LEDD, levodopa‐equivalent daily dose; H&Y, Hoehn and Yahr stage; PDQ, Parkinson's Disease Questionnaire.

Associations between key clinical characteristics at prior visits and treatment practices at follow‐up visit are presented in Table 2. Falls (*β* = 0.44, 95% confidence interval [CI]: [0.36, 0.52], *P* < 0.001), H&Y Stage 3 vs. 1–2 (*β* = 0.47, 95% CI: [0.38, 0.56], *P* < 0.001), H&Y stage 4–5 vs.1–2 (*β* = 0.62, 95% CI: [0.47, 0.76], *P* < 0.001), and PDQ‐39 Mobility subscale (*β* = 0.47, 95% CI: [0.39, 0.55], *P* < .001) were positively associated with a higher likelihood of PT utilization. After a fall, PT use ranged from 32% to 88% across centers. Mental health services utilization was predicted by PDQ‐39 emotional subscale (*β* = 1.74, 95% CI [1.50, 1.98], *P* < 0.001), with the use of mental health services ranging from 25% to 100% across centers for PDQ‐39 emotional subscale >9.

## Discussion

In this study, we analyzed a large clinical dataset to evaluate real‐world treatment patterns in PD. Across an international sample of COEs, we found high variability in how levodopa, dopamine agonists, mental health services, DBS, and PT were utilized. However, patients with greater disease severity were more likely to receive PT and mental health treatment at a subsequent visit, confirming the use of reactive allied health services. Our results suggest that COEs have similar standards of care for mental health services and PT referral, but that treatment strategies for PD are generally inconsistent across the patient population and, overall, underutilized.

This unique study described how treatment practices in PD differ globally across care centers. The substantial variation observed was more than expected and not explained by measured patient factors or center practices. We hypothesize that additional, unmeasured factors, such as differences in physician practice, locally available resources, patient preferences, and additional clinical heterogeneity not captured by traditional measures, may have contributed to observed differences. However, more concerning is that even at centers of expert care the variability in care, particularly around standard of care practices such as PT treatment after a fall, could also indicate inequities in care and reduced quality of care.^17^ In the community neurology setting, this variability may be even more striking. Some researchers argue that patients with PD have inconsistent responses to treatment and progress at different rates, thus requiring individualized care.^18^ Furthermore, non‐PD‐related factors, such as patients' lifestyle, comorbidities, and age, can also influence how PD is managed. Although our results suggest that the real‐world treatment of PD is complex, it is nonetheless imperative that practical treatment guidelines are developed and assessed so that all people with PD receive standardized, equitable, and high‐quality care regardless of where and from whom they receive treatment.

There are numerous factors that influence the prescribing behavior of healthcare providers. These include drug‐related factors (effectiveness, safety, personal experience, formulary status, ease of use, and cost), direct factors (drug availability and guidelines), and indirect factors (drug samples, direct to consumer advertising, and pharmaceutical sponsored educational material).^19^, ^20^ All of these factors likely played a role, to varying degrees based on the provider and center, in the wide range in frequency observed for dopamine agonist use in early PD.

In addition, given that treatment approaches are partially influenced by a clinician's discretion, implicit biases may impact how certain patients are treated. A recent report provides a telling example of how implicit biases can affect care in PD. Harris et al. identified that biases and stereotypes are associated with the facial expressivity of African American patients, which, in turn, impede practitioners from identifying hypomimia impairment and treatment of motor symptoms.^21^ Implicit biases have been shown to contribute to the health disparities and inequalities consistently noted in the treatment of marginalized populations.^22^ Research indicates that Black patients with PD have significantly worse PDQ‐39 scores and are less likely to receive medication treatment or PT compared to their White counterparts.^17^, ^23^ Indeed, previous findings from the PF‐POP show that the mean LEDD was significantly higher in White patients than Black patients.^17^ Additionally, despite all groups having PDQ‐39 emotional subscores ≥ 10, White patients were treated with antidepressants more often than Black, Hispanic, and Asian patients. Thus, to establish optimal practices for all PD patient populations, it is imperative to understand why we see variations in care, measure the role of implicit biases, and eliminate these biases.

Moreover, there may be treatment disparities that stem from variances in the healthcare settings where patients with PD seek care. In the United States, approximately 90% of PD patients consult with the following clinicians: movement disorder specialists (9.1%), general neurologists (50.9%), primary care physicians, or receive no physician consultation at all (40%).^24^ Urban areas tend to favor movement disorder specialists, whereas rural areas lean toward primary care physicians and general neurologists. Rural PD management often relies on levodopa alone, lacking the diverse and advanced therapies offered by movement disorder specialists, which could result in worse outcomes.^25^, ^26^, ^27^ This indicates that the underutilization of care options observed in our sample may be even more pronounced for most individuals with PD who do not receive care from movement disorder specialists or live in rural areas.

The utilization of mental health services was associated with worse PDQ‐39 emotional subscale scores. We found that 20% more patients were treated with antidepressants or visited a psychiatrist or psychologist when more symptoms of emotional distress were expressed. As other studies have reported that less than half of individuals with PD initiate antidepressants^28^ and only 6% visit a psychiatrist or psychologist,^24^ these results suggest that patients at COEs are more likely to receive mental health treatment than other patients in the general population, although rates of treatment are still suboptimal. A COE requirement is to have access to multidisciplinary care teams, which frequently consist of specialized psychiatrists and psychologists.^29^, ^30^ Higher mental health treatment rates at COEs may also be reflective of movement disorder specialists having additional awareness and education about the high prevalence of neuropsychiatric symptoms in PD and a greater likelihood of using nonmotor screening instruments in clinic than general neurologists or primary care physicians. Despite the improvement in provider knowledge, screening and access, unaddressed barriers to treatment remain, including low mental health literacy among patients and inadequate treatment options.^27^

In early PD, we found a wide range (13%–71%) of PT utilization across the centers. However, there is a growing body of evidence that early PT improves long‐term outcomes^31^ and supports greater utilization of PT even before a fall. We did find that the likelihood of PT utilization was higher in individuals with more falls and worsening H&Y stage. This was expected, given evidence from systematic reviews and meta‐analyses that PT can temporarily improve activities of daily living, motor functioning, and reduce fall risk in PD.^32^, ^33^, ^34^ Patients with PD may also benefit from specific exercise interventions, such as tai chi and balance training, which were associated with reduced fall rates over 6‐month and 12‐month follow‐up periods, respectively.^33^ Our findings corroborate an earlier report that analyzed a smaller POP sample and similarly found that PT referral increased in response to disease progression.^35^ Keus et al. administered questionnaires to individuals with PD in the Netherlands and identified an association between PT treatment rate and the number of falls in the previous year.^36^ There may be other factors predicting utilization of PT, however, such as age, sex, cost of care, insurance status, transportation barriers, and neurologist care.^26^, ^37^

As PD advances and becomes more complicated, invasive therapies such as DBS, pump therapies, and lesional therapies are often used. Recent guidelines from the European Academy of Neurology and the European Section of the Movement Disorders Society recommend that DBS should be offered to people with advanced PD if medications are not adequately controlling their fluctuations or tremor.^38^ We were unable to evaluate the use of pump therapy or lesional therapy due to small sample sizes. However, we observed wide variation in DBS use across centers. This finding again raises the concern that variations in care may, in part, reflect healthcare disparities, as there is substantial evidence that both women and minoritized groups are less likely to receive DBS.^39^

Surprisingly, worse PDQ‐39 mobility subscale or H&Y stage did not predict an increase in LEDD. In a previous study, H&Y stage was shown to predict total levodopa dose.^40^ Recent trials demonstrate that levodopa is the best treatment choice for patients with early PD, as it is associated with improved long‐term outcomes relative to levodopa‐sparing strategies and does not increase the prevalence of motor complications compared to delaying treatment.^41^, ^42^ However, our results raise the question of how this treatment is adjusted as PD progresses. Patients at COEs may not have warranted increased LEDD because their disease symptoms were mitigated by treatment with advanced therapies or nonpharmacological interventions, dose changes may have been too small to detect a difference, or they could have experienced bothersome side effects, including cognitive side effects, which increase with advancing disease. The implementation of standardized approaches to PD care that improve objective outcomes will help reduce variability in care and can help guide the general neurologist or primary care provider whom most patients are seeing.

Despite the large sample size and involvement of many centers, this study included a selective population of patients treated at academic centers who were predominantly White. We also could not account for every potential covariate or confounder during the analysis, as this was an observational study. Therefore, our results cannot infer causal relationships and cannot be generalized to the entire PD population. Furthermore, as in any real‐world study, there was missing data that could bias the findings.

We recommend a follow‐up qualitative research study to gain additional context into the underlying reasons for the observed variation in the utilization of treatment practices at COEs through interviews or focus groups of key stakeholders, including patients and families, community and specialty providers, and community service organizations. Additionally, a pragmatic, randomized‐controlled trial of common treatment practices can help understand how the utilization of mental health and PT affects long‐term patient outcomes. Future studies will need to account for the heterogeneity within PD, related and unrelated comorbidities in this older population, and include community centers in addition to specialty centers of care.

In summary, this large, observational study of PD treatment and outcomes underscores the large variability in PD practice across centers of excellence. Our findings highlight the pressing need for the development and implementation of standardized PD treatment guidelines to ensure the delivery of high‐quality, equitable care. Understanding the factors driving this variability in a broader PD population will be crucial in advancing quality of care, reducing inequities, and developing best practices. Furthermore, these findings reinforce the importance of empowering patients and families through PD education, increasing knowledge among all PD providers, shared decision‐making, and understanding patient preferences, implicit biases, and cultural context given this high variability in treatment practices.

## Author Roles

(1) Research project: A. Conception, B. Organization, C. Execution; (2) Statistical analysis: A. Design, B. Execution, C. Review and critique; (3) Manuscript preparation: A. Writing of the first draft, B. Review and critique.

N.D: 1C, 2C, 3A

T.K: 3A, 3B

A.C: 3A, 3B

J.C.B: 1B, 3B

T.L.D: 1C, 2C, 3B

H.L: 2B, 3B

S.L: 2B, 3B

A.N: 1B, 1C, 3B

M.N: 3B

M.R.R: 1C, 2C, 3B

A.R: 1C, 2C, 3B

C.M: 1A, 1B, 1C, 2A, 2C, 3B

## Disclosures

**Ethical Compliance Statement:** The study received approval from each of the participating centers' individual Institutional Review Boards (IRBs), and written informed consent was obtained from all participants. We confirm that we have read the journal's position on issues involved in ethical publication and affirm that this work is consistent with those guidelines.

**Funding Sources and Conflict of Interest:** Nabila Dahodwala, Thomas L. Davis, Hongliang Liu, Sheng Luo, Marilyn Neault, Miriam R. Rafferty, Adolfo Ramirez‐Zamora, and Connie Marras received grants or honoraria from the Parkinson's Foundation to conduct this study. James C. Beck and Anna Naito were employees of the Parkinson's Foundation.

**Financial Disclosures for the Previous 12 Months:** Nabila Dahodwala has received honoraria from the Parkinson Study Group; research grants from the NIH, MJFF, and the Parkinson's Foundation; and expert testimony fees from MotleyRice. Theodore Kapogiannis and Amanda Cruz have no disclosures to report. James C. Beck received research funding from the NIH and MJFF and was employed by the Parkinson's Foundation. Thomas L. Davis has received income for consultancy from AbbVie Pharmaceutical and research funding from the Parkinson's Foundation. Hongliang Liu has no disclosures to report. Sheng Luo received consulting or advisory board membership with honoraria from the National Institutes of Health, IQVIA, BIAL Biotech, Parkinson Study Group, Huntington Study Group, International Parkinson and Movement Disorder Society; grants/research from the National Institutes of Health, CHDI Foundation/Management, Inc., International Parkinson and Movement Disorder Society, and Parkinson's Foundation; and a salary from Duke University. Anna Naito was employed by the Parkinson's Foundation. Marilyn Neault received consulting honoraria from the Parkinson's Foundation. Miriam R. Raferty has received research grants from the Parkinson's Foundation. Adolfo Ramirez‐Zamora received consulting honoraria from Medtronic, Boston Scientific, Iota Inc, AbbVie, and Guidepoint. Connie Marras has received grants from MJFF, the Parkinson's Foundation, the Weston Brain Institute, and the Academic Health Sciences Alternate Funding Plan Innovation Fund; and salary support from the University Health Network and the Mayvon Foundation.

## Supporting information


**Fig. S1.** Locations of participating Parkinson Foundation Centers of Excellence.

**TABLE S1.** Dose/frequency of primary practices of interest by center (n = 31) among those with disease duration from diagnosis ≤ 5 years.
**TABLE S2.** Dose/frequency of primary practices of interest by center (n = 31) among those with disease duration from diagnosis >5 years.

## Data Availability

The data that support the findings of this study are available on request from the corresponding author. The data are not publicly available due to privacy or ethical restrictions.
