# Supplementary material for: Longitudinal Study of Treatment Variability for Parkinson's Disease across Specialized Centers
Source: Mov Disord Clin Pract. 2025 Jul 15;12(12):2207–16. doi: 10.1002/mdc3.70232 (PMC12715345; doi:10.1002/mdc3.70232)
Supplement: Supplementary file 1 — Fig. S1. Locations of participating Parkinson Foundation Centers of Excellence. [file MDC3-12-2207-s002.docx]

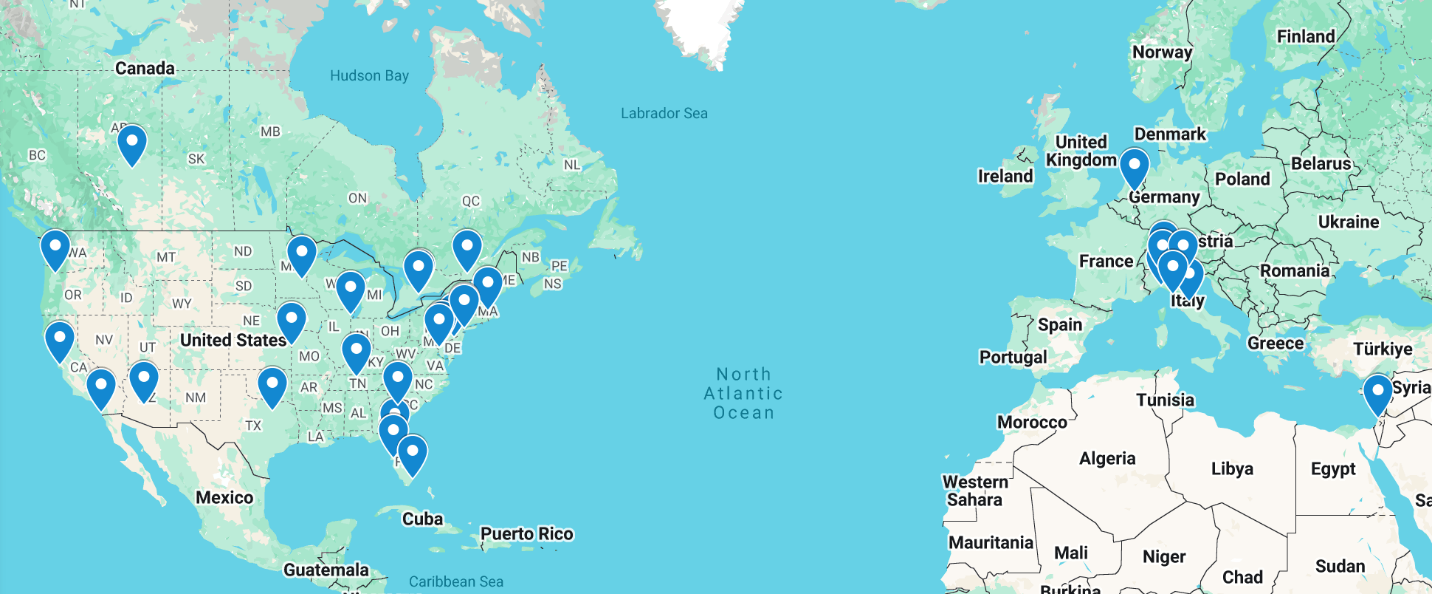


Supplementary Figure 1. Locations of participating Parkinson Foundation Centers of Excellence
