# Supplementary material for: Longitudinal Study of Treatment Variability for Parkinson's Disease across Specialized Centers
Source: Mov Disord Clin Pract. 2025 Jul 15;12(12):2207–16. doi: 10.1002/mdc3.70232 (PMC12715345; doi:10.1002/mdc3.70232)
Supplement: Supplementary file 2 — TABLE S1. Dose/frequency of primary practices of interest by center (n = 31) among those with disease duration from diagnosis ≤ 5 years. TABLE S2. Dose/frequency of primary practices of interest by center (n = 31) among those with disease duration from diagnosis >5 years. [file MDC3-12-2207-s001.docx]

Supplementary Tables

| Supplementary Table 1: Dose/Frequency of primary practices of interest by center (n=31) among those with disease duration from diagnosis <=5 years | | | | | | | | | | |
| --- | --- | --- | --- | --- | --- | --- | --- | --- | --- | --- |
| Center # | Using Levodopa, n (%) | OR (95%CI)^1^ | Using Dopamine Agonist, n (%) | OR (95%CI)^1^ | Using Physical Therapy, n (%) | OR (95%CI)^1^ | Using Psychiatrist or Psychologist, n (%) | OR (95%CI)^1^ | Using Deep Brain Stimulation, n (%) | OR (95%CI)^1^ |
| 1 | 149 (72) | 0.75 (0.55-1.03) | 69 (34.2) | 1.08 (0.79-1.48) | 79 (37.4) | 1.18 (0.89-1.57) | 4 (13.3) | 1.18 (0.41-3.37) | 5 (2.4) | 1.44 (0.59-3.49) |
| 2 | 165 (80.5) | 1.16 (0.81-1.66) | 25 (12.2) | 0.3 (0.2-0.47)*** | 60 (29.3) | 0.79 (0.58-1.07) | 2 (22.2) | 4.45 (0.9-21.99) | 1 (0.5) | 0.35 (0.05-2.31) |
| 3 | 279 (88) | 2 (1.41-2.83) | 71 (23.8) | 0.73 (0.55-0.97)* | 155 (48.6) | 1.69 (1.35-2.12)*** | 10 (11.5) | 0.96 (0.49-1.91) | 3 (0.9) | 0.61 (0.2-1.87) |
| 4 | 180 (66.4) | 0.62 (0.48-0.82)*** | 111 (42.4) | 1.44 (1.11-1.89)** | 138 (50) | 2.05 (1.61-2.62)*** | 2 (4) | 0.3 (0.07-1.21) | 2 (0.7) | 0.41 (0.11-1.61) |
| 5 | 221 (62.8) | 0.47 (0.37-0.6)*** | 116 (33) | 1.08 (0.85-1.38) | 143 (40.6) | 1.36 (1.09-1.7)** | 13 (14.1) | 2.11 (1.13-3.94)* | 3 (0.9) | 0.55 (0.18-1.67) |
| 6 | 188 (74.9) | 0.99 (0.73-1.34) | 118 (47.2) | 1.82 (1.39-2.39)*** | 109 (43.3) | 1.58 (1.23-2.04)*** | 7 (31.8) | 3.59 (1.38-9.37)** | 9 (3.6) | 2.23 (1.11-4.45)* |
| 7 | 275 (76.6) | 1.08 (0.83-1.4) | 132 (36.8) | 1.12 (0.88-1.42) | 86 (23.9) | 0.61 (0.48-0.79)*** | 4 (4.7) | 0.33 (0.12-0.89)* | 8 (2.2) | 1.26 (0.61-2.58) |
| 8 | 140 (59.3) | 0.5 (0.38-0.66)*** | 58 (24.8) | 0.58 (0.42-0.79)*** | 59 (25.1) | 0.73 (0.54-0.98)* | 6 (16.2) | 1.47 (0.59-3.63) | 2 (0.9) | 0.5 (0.13-1.96) |
| 9 | 296 (86.8) | 1.97 (1.43-2.72)*** | 154 (45.2) | 1.8 (1.42-2.28)*** | 91 (26.7) | 0.68 (0.53-0.86)** | 12 (9.6) | 0.77 (0.41-1.44) | 6 (1.8) | 1.01 (0.45-2.3) |
| 10 | 266 (70.7) | 0.72 (0.57-0.92)** | 117 (31.1) | 0.91 (0.72-1.17) | 148 (39.4) | 1.26 (1.01-1.56)* | 14 (24.1) | 2.19 (1.15-4.17)* | 11 (2.9) | 1.79 (0.95-3.37) |
| 11 | 155 (75.2) | 0.78 (0.56-1.08) | 76 (36.9) | 1.46 (1.08-1.98)* | 84 (41.2) | 1.28 (0.97-1.7) | 4 (8.7) | 0.81 (0.29-2.28) | 4 (2) | 1.33 (0.5-3.56) |
| 12 | 158 (62.2) | 0.46 (0.35-0.6)*** | 116 (45.7) | 1.91 (1.46-2.5)*** | 49 (19.3) | 0.47 (0.35-0.65)*** | 0 (0) | - | 4 (1.6) | 1.05 (0.39-2.8) |
| 13 | 94 (81.7) | 1.2 (0.74-1.93) | 33 (31.4) | 1.08 (0.71-1.65) | 58 (48.7) | 1.78 (1.24-2.55)** | 2 (4.5) | 0.48 (0.12-1.93) | 1 (0.9) | 0.55 (0.08-3.7) |
| 14 | 272 (78.8) | 1.06 (0.8-1.4) | 73 (21.2) | 0.58 (0.44-0.77)*** | 106 (30.7) | 0.86 (0.68-1.08) | 3 (5.8) | 0.6 (0.19-1.92) | 4 (1.2) | 0.76 (0.28-2.01) |
| 15 | 228 (87.4) | 2.27 (1.57-3.29)*** | 101 (38.7) | 1.24 (0.95-1.62) | 72 (27.6) | 0.78 (0.59-1.03) | 1 (2) | 0.15 (0.02-1) | 9 (3.5) | 2.09 (1.05-4.17)* |
| 16 | 33 (70.2) | 0.7 (0.37-1.31) | 17 (36.2) | 1.19 (0.65-2.18) | 12 (25.5) | 0.68 (0.35-1.29) | - | - | 0 (0) | - |
| 17 | 159 (79.1) | 1.01 (0.71-1.43) | 68 (33.8) | 1.21 (0.88-1.64) | 73 (36.1) | 1.02 (0.76-1.36) | 8 (13.6) | 1.1 (0.51-2.37) | 7 (3.5) | 2.25 (1.04-4.86)* |
| 18 | 171 (64.5) | 0.46 (0.35-0.6)*** | 45 (17.4) | 0.46 (0.33-0.64)*** | 73 (26.7) | 0.64 (0.49-0.84)** | 3 (3.6) | 0.24 (0.08-0.78)* | 8 (3) | 1.73 (0.83-3.6) |
| 19 | 114 (84.4) | 1.86 (1.16-2.99)* | 15 (11.4) | 0.22 (0.13-0.38)*** | 65 (48.1) | 2.06 (1.46-2.89)*** | 0 (0) | - | 1 (0.7) | 0.43 (0.06-2.87) |
| 20 | 202 (82.4) | 1.59 (1.13-2.24)** | 62 (25.5) | 0.63 (0.46-0.86)** | 74 (29.7) | 0.86 (0.65-1.13) | 13 (20.6) | 2.33 (1.24-4.4)** | 4 (1.6) | 0.95 (0.36-2.54) |
| 21 | 117 (65.4) | 0.56 (0.41-0.78)*** | 40 (22.5) | 0.55 (0.38-0.8)** | 37 (20.9) | 0.5 (0.34-0.71)*** | 7 (12.7) | 1 (0.44-2.3) | 1 (0.6) | 0.34 (0.05-2.26) |
| 22 | 105 (86.1) | 1.68 (1-2.82) | 16 (15.7) | 0.39 (0.22-0.66)*** | 48 (44.9) | 1.45 (0.99-2.12) | 20 (19) | 2.4 (1.41-4.07)** | 0 (0) | - |
| 23 | 83 (73.5) | 0.74 (0.48-1.14) | 21 (18.6) | 0.49 (0.3-0.8)** | 62 (54.9) | 1.89 (1.3-2.76)*** | 30 (26.5) | 3.18 (1.98-5.1)*** | 0 (0) | - |
| 24 | 70 (94.6) | 4.32 (1.6-11.64)** | 4 (8.2) | 0.18 (0.07-0.51)** | 54 (71.1) | 4.01 (2.45-6.57)*** | 24 (31.6) | 4.36 (2.56-7.43)*** | 4 (5.3) | 2.89 (1.04-8.06)* |
| 25 | 84 (83.2) | 1.09 (0.64-1.85) | 63 (71.6) | 5.95 (3.7-9.57)*** | 29 (24.4) | 0.54 (0.36-0.83)** | 3 (2.6) | 0.31 (0.1-0.97)* | 0 (0) | - |
| 26 | 68 (70.1) | 0.52 (0.33-0.82)** | 52 (58.4) | 3.46 (2.23-5.38)*** | 34 (34.3) | 0.98 (0.65-1.5) | 5 (5) | 0.78 (0.31-1.97) | 0 (0) | - |
| 27 | 80 (74.1) | 0.63 (0.4-1)* | 70 (70) | 6.36 (4.04-10.02)*** | 44 (35.5) | 0.83 (0.57-1.22) | 10 (8.1) | 0.66 (0.33-1.3) | 0 (0) | - |
| 28 | 77 (65.3) | 0.3 (0.19-0.45)*** | 41 (34.7) | 1.54 (1.01-2.33)* | 39 (33.1) | 0.68 (0.45-1.02) | 5 (4.2) | 0.3 (0.12-0.77)* | 0 (0) | - |
| 29 | 41 (95.3) | 4.24 (1.05-17.1)* | 24 (61.5) | 4.64 (2.4-8.97)*** | 19 (42.2) | 1.19 (0.65-2.16) | 3 (6.7) | 0.75 (0.22-2.5) | 0 (0) | - |
| 30 | 81 (84.4) | 1.15 (0.66-2.01) | 76 (81.7) | 11.33 (6.62-19.4)*** | 15 (13) | 0.24 (0.14-0.41)*** | 14 (12.2) | 1.41 (0.75-2.64) | 0 (0) | - |
| 31 | 52 (86.7) | 1.68 (0.8-3.53) | 1 (1.7) | 0.04 (0.01-0.24)*** | 28 (46.7) | 1.42 (0.86-2.36) | 15 (25) | 3.09 (1.64-5.81)*** | 3 (5) | 2.93 (0.91-9.38) |
| Total | 4603 (75.5) |  | 1985 (33.3) |  | 2143 (34.7) |  | 244 (12.1) |  | 100 (1.6) |  |

| Supplementary Table 2: Dose/Frequency of primary practices of interest by center (n=31) among those with disease duration from diagnosis >5 years | | | | | | | | | | | | | |
| --- | --- | --- | --- | --- | --- | --- | --- | --- | --- | --- | --- | --- | --- |
| Center # | Using Levodopa, n (%) | OR (95%CI)^1^ | | Using Dopamine Agonist, n (%) | OR (95%CI)^1^ | Using Physical Therapy, n (%) | OR (95%CI)^1^ | Using Psychiatrist or Psychologist, n (%) | | OR (95%CI)^1^ | Using Deep Brain Stimulation, n (%) | OR (95%CI)^1^ | |
| 1 | 350 (96.7) | 1.42 (0.8-2.55) | | 120 (33.6) | 0.48 (0.38-0.6)*** | 160 (44.2) | 1.22 (0.98-1.51) | 2 (9.1) | | 0.71 (0.17-2.95) | 149 (41.2) | 3.77 (2.98-4.76)*** | |
| 2 | 276 (95.2) | 0.82 (0.47-1.42) | | 90 (31.2) | 0.45 (0.35-0.58)*** | 94 (32.4) | 0.69 (0.54-0.89)** | 1 (20) | | 2.01 (0.23-17.5) | 7 (2.4) | 0.13 (0.06-0.28)*** | |
| 3 | 338 (97.1) | 1.62 (0.86-3.05) | | 149 (44.6) | 0.8 (0.64-1.01) | 167 (48) | 1.26 (1.01-1.56)* | 10 (12.2) | | 1 (0.51-1.99) | 59 (17) | 1.18 (0.88-1.58) | |
| 4 | 285 (92.8) | 0.64 (0.41-1) | | 131 (43.5) | 0.72 (0.57-0.91)** | 150 (48.9) | 1.45 (1.15-1.83)** | 4 (10) | | 0.91 (0.32-2.53) | 54 (17.8) | 1.07 (0.78-1.45) | |
| 5 | 237 (96) | 1.09 (0.58-2.07) | | 122 (49.4) | 0.96 (0.74-1.24) | 138 (55.9) | 1.85 (1.43-2.39)*** | 5 (13.5) | | 1.42 (0.55-3.67) | 25 (10.2) | 0.6 (0.39-0.91)* | |
| 6 | 328 (95.3) | 1.07 (0.64-1.8) | | 198 (59.1) | 1.34 (1.07-1.69)* | 164 (47.3) | 1.4 (1.13-1.74)** | 7 (16.7) | | 1.57 (0.69-3.57) | 72 (20.7) | 1.26 (0.95-1.66) | |
| 7 | 318 (92.7) | 0.67 (0.43-1.02) | | 157 (45.6) | 0.8 (0.64-1)* | 98 (28.6) | 0.59 (0.46-0.75)*** | 4 (5.1) | | 0.36 (0.13-0.98)* | 75 (21.9) | 1.38 (1.05-1.82)* | |
| 8 | 193 (93.7) | 0.67 (0.38-1.19) | | 93 (45.1) | 0.79 (0.59-1.04) | 63 (30.9) | 0.67 (0.5-0.9)** | 2 (10) | | 0.97 (0.23-4.07) | 55 (27.2) | 2.03 (1.47-2.81)*** | |
| 9 | 371 (96.4) | 1.43 (0.83-2.45) | | 188 (48.8) | 0.9 (0.73-1.11) | 139 (36.1) | 0.84 (0.68-1.04) | 13 (12.1) | | 0.98 (0.54-1.8) | 88 (22.9) | 1.46 (1.13-1.89)** | |
| 10 | 251 (98) | 2.68 (1.12-6.4)* | | 104 (40.9) | 0.66 (0.51-0.85)** | 133 (52) | 1.63 (1.27-2.09)*** | 18 (36.7) | | 3.7 (1.99-6.88)*** | 76 (29.9) | 2.24 (1.68-2.98)*** | |
| 11 | 195 (93.3) | 0.6 (0.35-1.04) | | 109 (51.9) | 1.16 (0.88-1.53) | 100 (47.6) | 1.22 (0.93-1.6) | 5 (8.9) | | 0.9 (0.36-2.25) | 20 (9.5) | 0.62 (0.39-0.98)* | |
| 12 | 208 (87.4) | 0.3 (0.2-0.46)*** | | 139 (58.9) | 1.38 (1.06-1.8)* | 56 (23.4) | 0.45 (0.34-0.61)*** | - | | - | 47 (19.7) | 1.29 (0.92-1.8) | |
| 13 | 133 (96.4) | 1.2 (0.5-2.91) | | 61 (49.6) | 0.98 (0.69-1.4) | 90 (62.1) | 2.21 (1.58-3.09)*** | 5 (8.6) | | 0.78 (0.31-1.97) | 45 (31.9) | 2.79 (1.93-4.04)*** | |
| 14 | 228 (95.4) | 0.87 (0.47-1.6) | | 113 (47.5) | 0.93 (0.71-1.21) | 93 (38.9) | 0.88 (0.67-1.14) | 1 (3.7) | | 0.31 (0.04-2.16) | 20 (8.5) | 0.55 (0.35-0.87)* | |
| 15 | 283 (95.9) | 1.25 (0.69-2.24) | | 159 (53.9) | 1.03 (0.81-1.31) | 93 (31.5) | 0.76 (0.59-0.97)* | 0 (0) | | - | 83 (28.3) | 1.81 (1.38-2.38)*** | |
| 16 | 59 (100) | - | | 23 (39) | 0.64 (0.38-1.06) | 19 (32.2) | 0.73 (0.43-1.24) | - | | - | 17 (28.8) | 2.07 (1.17-3.66)* | |
| 17 | 167 (96.5) | 1.41 (0.63-3.16) | | 87 (50.6) | 0.98 (0.72-1.32) | 56 (32.4) | 0.7 (0.51-0.96)* | 7 (15.2) | | 1.48 (0.65-3.37) | 41 (24.3) | 1.7 (1.18-2.44)** | |
| 18 | 331 (90.9) | 0.49 (0.33-0.72)*** | | 101 (29.4) | 0.41 (0.32-0.52)*** | 124 (33.5) | 0.72 (0.58-0.89)** | 5 (4.5) | | 0.35 (0.14-0.86)* | 85 (23.2) | 1.61 (1.24-2.1)*** | |
| 19 | 63 (98.4) | 3.24 (0.48-22.06) | | 26 (40.6) | 0.59 (0.36-0.97)* | 42 (65.6) | 3.29 (1.97-5.47)*** | - | | - | 5 (7.8) | 0.35 (0.14-0.86)* | |
| 20 | 297 (95.5) | 1.12 (0.65-1.93) | | 124 (40.1) | 0.62 (0.49-0.78)*** | 93 (29.8) | 0.65 (0.51-0.83)*** | 12 (16.7) | | 1.57 (0.82-3) | 87 (28.2) | 1.88 (1.43-2.45)*** | |
| 21 | 127 (91.4) | 0.55 (0.3-1) | | 70 (50.7) | 0.99 (0.71-1.38) | 44 (32.1) | 0.7 (0.49-1) | 4 (11.4) | | 0.88 (0.31-2.51) | 8 (5.8) | 0.29 (0.14-0.58)*** | |
| 22 | 104 (94.5) | 0.99 (0.44-2.24) | | 42 (47.2) | 0.88 (0.58-1.34) | 51 (48.1) | 1.26 (0.86-1.84) | 21 (20) | | 2.41 (1.43-4.05)*** | 0 (0) | - | |
| 23 | 78 (94) | 0.88 (0.36-2.17) | | 26 (31.3) | 0.44 (0.27-0.7)*** | 47 (56.6) | 1.67 (1.08-2.57)* | 20 (24.1) | | 2.31 (1.34-3.99)** | 8 (9.6) | 0.6 (0.29-1.24) | |
| 24 | 129 (97.7) | 2.34 (0.76-7.19) | | 27 (27.3) | 0.4 (0.26-0.62)*** | 82 (61.2) | 2.16 (1.53-3.06)*** | 38 (28.4) | | 3.78 (2.46-5.82)*** | 22 (16.4) | 1.08 (0.68-1.72) | |
| 25 | 72 (94.7) | 0.94 (0.34-2.57) | | 50 (84.7) | 6.65 (3.3-13.4)*** | 40 (44.9) | 1.03 (0.68-1.58) | 2 (2.2) | | 0.23 (0.06-0.9)* | 8 (9) | 0.63 (0.3-1.31) | |
| 26 | 233 (91.7) | 0.58 (0.36-0.93)* | | 144 (57.8) | 1.5 (1.15-1.95)** | 109 (42.9) | 1.07 (0.83-1.39) | 16 (6.3) | | 0.66 (0.38-1.14) | 20 (8.1) | 0.48 (0.3-0.76)** | |
| 27 | 72 (97.3) | 2.32 (0.58-9.22) | | 53 (81.5) | 5.01 (2.69-9.34)*** | 37 (47.4) | 1.25 (0.8-1.96) | 18 (23.1) | | 2.45 (1.37-4.38)** | 17 (22.1) | 1.46 (0.83-2.54) | |
| 28 | 82 (94.3) | 0.89 (0.35-2.24) | | 45 (51.7) | 1.4 (0.9-2.16) | 29 (33.3) | 0.58 (0.37-0.92)* | 1 (1.1) | | 0.09 (0.01-0.63)* | 7 (8.3) | 0.63 (0.29-1.36) | |
| 29 | 142 (94) | 0.87 (0.43-1.74) | | 103 (76.3) | 3.61 (2.41-5.4)*** | 52 (34.2) | 0.71 (0.51-1.01) | 14 (9.2) | | 0.91 (0.5-1.66) | 41 (27) | 2.19 (1.49-3.2)*** | |
| 30 | 80 (100) | - | | 63 (90) | 10.51 (4.88-22.63)*** | 25 (29.4) | 0.55 (0.35-0.88)* | 15 (17.6) | | 1.84 (1-3.38) | 3 (3.5) | 0.2 (0.06-0.62)** | |
| 31 | 38 (92.7) | 0.63 (0.2-2.01) | | 9 (22) | 0.29 (0.14-0.6)*** | 15 (36.6) | 0.73 (0.39-1.37) | 6 (14.6) | | 1.45 (0.61-3.48) | 7 (17.1) | 1.23 (0.55-2.76) | |
| Total | 6068 (94.7) | |  | 2926 (47.1) |  | 2603 (40.4) |  | 256 (12.6) |  | | 1251 (19.5) | |  |
